# *Salmonella* Bloodstream Infections

**DOI:** 10.3390/tropicalmed8110487

**Published:** 2023-10-27

**Authors:** Micah J. Worley

**Affiliations:** Department of Biology, University of Louisville, Louisville, KY 40292, USA; micah.worley@louisville.edu; Tel.: +1-502-852-0966

**Keywords:** *Salmonella*, bacteremia, sepsis, bloodstream infections, serovars, pathogenicity islands

## Abstract

*Salmonella* is a major foodborne pathogen of both animals and humans. This bacterium is responsible for considerable morbidity and mortality world-wide. Different serovars of this genus cause diseases ranging from self-limiting gastroenteritis to a potentially fatal systemic disease known as enteric fever. Gastrointestinal infections with *Salmonella* are usually self-limiting and rarely require medical intervention. Bloodstream infections, on the other hand, are often fatal even with hospitalization. This review describes the routes and underlying mechanisms of the extraintestinal dissemination of *Salmonella* and the chronic infections that sometimes result. It includes information on the pathogenicity islands and individual virulence factors involved in systemic dissemination as well as a discussion of the host factors that mediate susceptibility. Also, the major outbreaks of invasive *Salmonella* disease in the tropics are described.

## 1. Introduction

### 1.1. Background

The genus *Salmonella* is composed of two species, *Salmonella bongori*, which is a commensal of reptiles but can cause gastroenteritis in humans, and *Salmonella enterica.* The latter is divided into six subspecies and remarkably nearly 3000 serovars [1]. These serovars are classified into two groups. The members of the typhoidal group, *Salmonella typhi*, *Salmonella sendai* and *Salmonella paratyphi*, are human-restricted and cause systemic illness, namely typhoid and paratyphoid fever. *S.* Typhimrium, *S.* Enteritidis and *S.* Dublin are some of the prevalent members of the non-typhoidal group that cause illness in humans. Invasive disease with non-typhoidal serovars, mostly Typhimurium and Enteritidis, is on the rise and is especially troublesome in sub-Saharan Africa. The impact of invasive *Salmonella* on public health is exacerbated by the emergence of multi-drug-resistant strains, which has nullified the effectiveness of most antibiotics, and by the lack of a licensed vaccine for non-typhoidal *Salmonella* [2,3].

### 1.2. Epidemiology

Globally, *Salmonella* is, notably, estimated to infect between 200 million to over 1 billion people per year [4]. The total number of infections is an estimate because so many cases go unreported. Typhoidal organisms are a major public health threat, infecting 14 million people per year, which leads to 110,000 deaths [5]. Invasive disease with non-typhoidal *Salmonella* is most problematic in sub-Saharan Africa, where it kills about 680,000 people per year [6].

### 1.3. Course of Infection

Fecal–oral transmission is the natural route of *Salmonella* infection. It is typically inadvertently ingested orally along with contaminated food or water. Gastroenteritis symptoms resulting from infection with non-typhoidal serovars that typically manifest within 8 to 72 h include diarrhea, nausea, vomiting, abdominal cramping and fever. The infection is usually self-limiting with the fever resolving within 48–72 h and the diarrhea within 4–10 days [7]. In otherwise healthy individuals, fewer than 5% of those infected will develop bacteremia. Those that are co-infected with malaria or HIV or are otherwise immunocompromised are at heightened risk. Bloodstream infections with non-typhoidal *Salmonella* are very serious, with a 20% fatality rate even with hospitalization [6]. Typhoidal serovars typically do not cause as much gut inflammation, but bloodstream infections are the norm with these serovars. The spleen and liver, which filter the blood, are often colonized. From the liver, the bacteria can spread to the gall bladder. In some cases, within systemic organs, the infection can become asymptomatic. With such infections, intermittent fecal shedding following the return of the bacteria to the gastrointestinal tract through the lymphatics and/or bile duct can occur for, in some cases, the life of the host.

## 2. Extraintestinal Dissemination

### 2.1. The Role of Pathogenicity Islands in Extraintestinal Dissemination

Nascent *Salmonella* began evolving into a pathogen after diverging 100–150 million years ago from a common ancestor with *Escherichia coli* [8]. One of the distinguishing features of *Salmonella* is contiguous regions of the genome, without corresponding ones in *E. coli*, that contain virulence genes, termed pathogenicity islands. They are of considerable interest for understanding the extraintestinal dissemination of *Salmonella* [9]. 

Researchers have identified and at least partially characterized twenty-three *Salmonella* pathogenicity islands [10]. The ones with established or presumed roles in regulating the systemic spread of *Salmonella* are discussed below. *Salmonella* pathogenicity island-1 and -2 encode two distinct type III secretion systems that secrete over 40 effectors into host cells. These virulence factors manipulate various cellular processes in ways that benefit the pathogen and largely dictate the course of infection. While numerous functions have been ascribed to them, many of them either induce or attenuate the host’s inflammatory response. The action and timing of the stimulation or suppression of this arm of the immune system is key to determining if the gastrointestinal tract is successfully colonized and whether the infection progresses beyond it.

*Salmonella* pathogenicity island-1-associated genes are primarily, although not exclusively, involved in promoting the invasion of host cells and the induction of inflammation [11,12]. The host intends for an inflammatory response to be protective, but *Salmonella* exacerbates it with pathogenicity island-1-associated virulence factors to aid the establishment of infection [13,14]. It enables the pathogen to overcome colonization resistance by reducing competition in the highly competitive environment found in the lumen of the gut. The reactive oxygen species generated yield new carbon sources and a new respiratory electron acceptor in tetrathionate that the resident microflora cannot use, thus enabling the outgrowth of *Salmonella* [15,16].

*Salmonella* pathogenicity island-2, on the other hand, mostly facilitates intracellular survival and proliferation but can also manipulate the adhesive and migratory properties of infected phagocytes [17,18,19,20,21,22,23,24]. This region of the genome is composed of four operons that contain over 40 genes, including the structural components of the type III secretion system, a specialized chaperone and seven effectors [17,18,25]. It also encodes a two-component regulator called SsrAB. SsrA is the sensor kinase that responds to an unknown intracellular cue and SsrB is its cognate transcription factor that activates gene expression within *Salmonella* pathogenicity island-2. It also, interestingly, coordinately induces the expression of type III effectors located outside of *Salmonella* pathogenicity island-2 [26]. These unlinked effectors were acquired independently of *Samonella* pathogenicity island-2, but regardless are secreted by its type III secretion system, which may have interesting evolutionary implications for virulence and gene regulation [27]. The *Salmonella* pathogenicity island-2 type III secretion system injects over 20 effectors through the vacuolar membrane surrounding intracellular *Salmonella* into the infected host cell’s cytosol [28].

In addition to facilitating intracellular growth, *Salmonella* pathogenicity island-2-associated effectors play a role in regulating the adhesive and migratory properties of infected phagocytes [20,21,22,24]. It is interesting to consider that while the type III secretion system harbored by *Salmonella* pathogenicity island-2 facilitates intracellular growth in vitro, it has a modest 0- to 5-fold effect over 24 h [17,18,19,29,30,31]. While there are multiple sequential rounds of cellular infection within a host, it is still curious that mutants that cannot secrete any *Salmonella* pathogenicity island-2 effectors are completely avirulent in animals. In fact, not a single *Salmonella* cell lacking a functional *Salmonella* pathogenicity island-2-encoded type III secretion system reaches the bloodstream of mice or chickens at any oral inoculum [31,32,33]. Perhaps the subversion of the adhesive and migratory properties of infected cells by type III secretion systems in a manner that promotes dissemination plays a major role in virulence [20,21,22].

*Salmonella* was traditionally thought to only deploy *Salmonella* pathogenicity island-2 in the systemic phase of disease, to facilitate intracellular survival and growth [17,18,19]. However, more recent studies have revealed that these effectors are expressed immediately in the gastrointestinal tract and secreted prior to penetration of the epithelium [20,21,22,34]. In addition to the temporal overlap in the expression of *Salmonella* pathogenicity island-1 and *Salmonella* pathogenicity island-2 effectors, there is also evidence of cooperative activity between them [35,36].

An essential component of being able to spread systemically is the ability to survive within macrophages, which *Salmonella* pathogenicity island-3 facilitates along with several other pathogenicity islands. *Salmonella* pathogenicity island-3 is a 17 Kb region of the genome that includes the important virulence genes *mgtCB*. MgtCB is a Mg2^+^ uptake system, which is necessary for the bacteria to overcome the severe Mg2^+^ deficiency imposed upon intra-vacuolar microbes by the host [37,38]. 

Breaching the intestinal epithelium is an important step in enabling systemic disease. *Salmonella* pathogenicity island-4 encodes a type I secretion system that mediates attachment to the apical face of the gastrointestinal epithelium, which is followed by bacterial internalization. This secretion system is composed of the five proteins SiiABCDF which secrete SiiE, which is a giant adhesin [39,40,41,42]. 

*Salmonella* pathogenicity island-5 is a 7 Kb region and includes five genes: *sopB*, *pipA*, *pipB*, *pipC* and *pipD*. The encoded proteins elicit inflammatory responses and fluid secretion [43,44]. Interestingly, they are differentially expressed and injected into host cells by the type III secretion systems harbored by *Salmonella* pathogenicity island-1 and *Salmonella* pathogenicity island-2 [44]. The 57 Kb *Salmonella* pathogenicity island-6 encodes a type 6 secretion system which has anti-bacterial activity that aids in overcoming colonization resistance, surviving within macrophages and facilitating systemic dissemination [45,46,47]. The largest pathogenicity island is SPI-7, which is a 134 Kb region. Most notably, it encodes the major Vi antigen and IV_B_ operon. These virulence factors attenuate innate immune responses and help the bacteria resist phagocyte-mediated killing [48]. They are thus likely important for systemic spread. SPI-7 is found in some strains of serovar Dublin and most strains of serovars Typhi and Paratyphi C [49].

Similar to *Salmonella* pathogenicity island-4, *Salmonella* pathogenicity island-9 mediates adhesion to the apical face of the gastrointestinal epithelium, which is necessary for some routes of extraintestinal dissemination [50]. Of the genes contained within this 32.8 Kb area of the genome, *prpZ* was shown to be important for *S*. Typhi survival within human macrophages [51]. The genes *pagC*, *pagD* and *msgA* are located within *Salmonella* pathogenicity island-11. These virulence factors contribute to the survival of *S*. Typhi in macrophages [52,53]. An encoded small RNA, RaoN promotes *S.* Typhimurium growth within macrophages [54].

### 2.2. What Comparative Genomics Tells Us about Extraintestinal Dissemination

All serovars of *Salmonella enterica* harbor pathogenicity islands 1–5 [38,55,56,57]. Of the remaining *Salmonella* pathogenicity islands (6–23), *S.* Typhimurium and *S.* typhi both have islands 6, 9, 11, 12, 13 and 16 [10,49,50,54,58,59,60,61,62,63,64,65]. Fourteen is specific to *S*. Typhimurium whereas 7, 8, 10, 15, 17 and 18 are missing in *S.* Typhimurium but are present in *S.* Typhi [10]. More research on these pathogenicity islands is needed to reveal the different strategies that different serovars and strains of *Salmonella* employ to either remain in the gut or spread beyond it.

As for individual type III effectors, a core set of them is conserved amongst many serovars of *Salmonella* including the prototypical typhoidal and non-typhoidal serovars *S.* Typhi and *S.* Typhimurium. This group includes those encoded by *pipA*, *pipB*, *pipB2*, *sifA*, *sipA*, *sipB*, *sipC*, *sipD*, *sopB*, *sopD*, *spiC*, *sptP*, *sseF*, *sseG*, *sseL*, *steA* and *steD* [66]. These genes may play roles more fundamental to *Salmonella* pathogenesis than facilitating extraintestinal dissemination. It is also possible that these proteins play a similar role in facilitating extraintestinal spread in different hosts. *S.* Typhimurium causes a systemic illness in its murine animal reservoir much like *S*. Typhi does in humans. It is interesting to note that while virulence genes such as these are generally noted as being present or absent, in some cases different alleles of the same gene can influence the course of infection [21,67]. In fact, a bioinformatics study found many instances of specific alleles of virulence genes being associated with either invasive or gastrointestinal disease [68]. Dominant alleles of *srfH*/*sseI*, *sopE*, *stfH*, *shdA*, *sifB*, *sopA*, *sseK2*, *bapA*, *siiE* and *sadA* were only found in strains associated with intestinal disease while those of *slrP*, *mgtB*, *fimH*, *srfA*, *steA*, *steC*, *zirS*, *fliC*, *sspH2*, *sseL* and *ratB* were found only in invasive stains [68]. Something that has not been considered yet is the potential for epistatic interactions amongst virulence factor alleles. It would be interesting to determine which combinations of various pro- and anti-inflammatory virulence factor alleles cause a strain to remain compartmentalized within the gastrointestinal tract or alternatively disseminate extraintestinally.

Seventeen type III effectors produced by *S*. Typhimurium are missing in most strains of *S*. Typhi and *S.* Paratyphi, including those encoded by *srfH*/*sseI*, *gogA*, *gogB*, *gtgA*, *gtgE*, *slrP*, *sopA*, *sopD2*, *spvB*, *spvC*, *spvD*, *srfJ*, *ssaJ*, *sseJ*, *sseK2* and *ssrA* [69]. The effectors encoded by *gtgA*, *srfH* and the *spv* operon, and perhaps some of the others, are important for systemic spread in mice [20,21,22]. Human-restricted serovars of *Salmonella* usually lack these genes, raising the possibility that they and invasive non-typhoidal strains have different mechanisms of extraintestinal spread. It is also possible that *S*. Typhi and *S.* Paratyphi possess functional analogs of these genes. 

### 2.3. Routes of Extraintestinal Dissemination

Despite its tremendous cost to human health, the routes, underlying mechanisms and relative importance of the many ways that *Salmonella* is known to traverse the mucosal barrier, disseminate to the systemic circulation and subsequently the internal organs, are incompletely understood. Conventionally, enteropathogens have been understood to transit from the lumen of the gut to the bloodstream by traveling passively through the lymphatic system [70,71,72]. *Salmonella* can colonize the lymphatic system by preferentially invading M cells, destroying them and then invading the adjacent enterocytes [73]. This induces the basolateral secretion of IL-8 from the gastrointestinal epithelium that attracts polymorphonuclear neutrophils. The tight junctions of the intestinal epithelium are essential for deterring the invasion of pathogens among other things [74]. The type III effector SipA induces the apical secretion of the chemoattractant hepoxilin A_3_ (HXA_3_) from the epithelium. The resulting gradient causes polymorphonuclear neutrophils to transmigrate from the lamina propria to the lumen of the gut through the paracellular space [75,76]. The tight junctions are further disrupted by the type III effector SpvB [77]. After invading the M cells, the bacteria can be carried to the mesenteric lymph nodes by phagocytes in order to generate an adaptive immune response. *Salmonella* can then drain through the thoracic duct into the bloodstream and go on to colonize the spleen and liver, which filter the blood.

There are numerous contradictory reports about the importance of disseminating through the lymphatic system to enteropathogens. *Salmonella* and *Yersinia* colonize the splenic and liver tissue of mice genetically devoid of Peyer’s patches and control mice with similar kinetics [78,79]. In another study, altering either the number or migratory properties of dendritic cells within the lymphatic system had no effect on bacteremia [80]. On the other hand, another report described extracellular *Salmonella* disseminating to the mesenteric lymph nodes independently of migratory host cells [81]. The possibility of extracellular *Salmonella* transiting through the thoracic duct into the blood would eliminate the need for intracellular *Salmonella* to manipulate the surface expression of the host receptors, integrins and selectins that control the movement of migratory host cells in the nodes. Sphingosine-1-phosphate receptor-1 is up-regulated in response to infection to trap infected cells within the nodes, presumably to guard against sepsis [82]. However, almost no *Salmonella* were observed to colonize the nodes of CCR7-deficient mice, suggesting that the bacteria are transported through the lymphatics by migratory host cells [80]. Interestingly, in CCR7-deficient mice there is no defect in bacterial translocation to the spleen and liver, supporting the idea that enteropathogens have alternative routes to spread from the gastrointestinal tract to the bloodstream [80].

A different pathway for enteropathogens to disseminate from the lumen of the gut to the blood involves the exploitation of CX3CR1^+^ phagocytes dispersed throughout the lamina propria. These sentinel cells send dendrites into the lumen of the gut to engage in antigen sampling [70]. Following internalization, *Salmonella* and perhaps other enteropathogens can cause the phagocytes that harbor them to enter the blood by inducing them to cross the blood vascular endothelium without disrupting the tight junctions, in the basal to apical direction [20,21,22,70,83]. Traversal of the endothelium in this manner is termed reverse transmigration with uninfected cells, and typically does not occur with infected ones [22]. Inducing these phagocytes to reverse transmigrate when infected with *Salmonella* requires the effector SpvC [22]. 

In yet another pathway, *Salmonella* can deploy *Salmonella* pathogenicity island-1 to invade the apical face of the gastrointestinal epithelium and then traffic to the basal side of the epithelium in a process that requires *Salmonella* pathogenicity island-2. The pathogen then exocytoses into the lamina propria where it is internalized by phagocytes [84]. *Salmonella* may be taking advantage of a host gastrointestinal epithelium antigen sampling pathway. The report that described this pathway used a mouse model for human gastroenteritis. It would be interesting to determine in a different model of disease, if *Salmonella* breached the mucosal barrier through this pathway and perhaps went on to disseminate to deeper tissue than the lamina propria. 

In the final known pathway, *Salmonella* pathogenicity island-2 can manipulate ß-catenin/Wnt signaling to increase the permeability of the endothelium of gut blood vessels [85]. No systemic spread was observed in transgenic mice expressing a ß-catenin allele within endothelial cells that was resistant to degradation. ß-catenin/Wnt signaling, however, affects many diverse cellular processes, complicating this interpretation [86]. 

### 2.4. The Roles of Pro- and Anti-Inflammatory Effectors in Extraintestinal Dissemination

Non-typhoidal *Salmonella* infections are fatal 20% of the time even with hospitalization if the bacteria enter the blood, but otherwise are usually mild [6]. Thus, understanding how the pathogen transitions from invoking and exacerbating inflammation to attenuating it, allowing for extraintestinal spread, could lead to new therapeutic intervention opportunities that reduce the likelihood of fatality. The properties of the major pro- and anti-inflammatory effectors of *Salmonella*, which are of special interest in understanding, and perhaps one day preventing, bloodstream infections are summarized in Table 1 and described below.

The induction and exacerbation of inflammation in the intestine is required for *Salmonella* to colonize a host. Accordingly, it possesses a suite of type III effectors that are pro-inflammatory. SopB, SopE and SopE2 are *Salmonella* pathogenicity island-1 effectors that short circuit endogenous host inflammation attenuation mechanisms. They function as guanine nucleotide exchange factors for the Rho family GTPases Rac and Cdc42, which stimulate MAPK and NF-κB signaling with an ensuant production of pro-inflammatory cytokines [97,100,101,117,118]. A signaling complex downstream of Toll-like receptors is also activated, that interestingly enhances the growth of *S*. Typhimurium in the gut but decreases its microbial spread to systemic sites [100]. 

The pro-inflammatory response induced by SopE, SopE2 and SopB is amplified by SopA. SopA ubiquitylates substrates with HECT-like E3 ligase activity. It has been reported to activate TRIM56 and TRIM65 to stimulate interferon genes and induce intestinal inflammation but in a separate report it was found to target these host proteins for proteasomal degradation [119,120]. This discrepancy warrants further investigation. The expression of a SopA E3 catalytic mutant renders *S*. Typhimurium less able to stimulate the transepithelial migration of PMNs [121]. SopB and SopD neutralize an anti-inflammatory pathway dependent upon AKT and Rab8, which is downstream of the Toll-like receptors that the host deploys to limit tissue damage following an infection [122,123].

*Salmonella* initially provokes and exacerbates host inflammation to facilitate colonization, but once established goes to great lengths to dampen it. Moderating inflammation in the gut may enable the pathogen to spread to systemic sites, which are nutrient-rich and less-competitive sites within the host, and may also promote a long-standing association with it. In fact, a number of type III effectors enhance intestinal disease but limit microbial spread to the bloodstream and internal organs [77,124,125]. The spatiotemporal regulation of the pro- and anti-inflammatory activities of the various *Salmonella* virulence factors may be one of the keys to understanding and perhaps one day modulating what type of disease is manifested upon infection.

SptP is a member of a group of virulence factors that directly antagonizes the activities of the pro-inflammatory factors, namely SopE and SopE2. SptP is a GTPase activating protein for CDC42, RAC1 and RhoA [106]. PipA, GtgA and GogA are related proteases that act on the NF-κB factors RELA and RELB [126]. At least one member of this set of virulence factors is found in all Salmonella isolates studied to date, revealing the importance of countering pro-inflammatory virulence factors in this fashion [126]. NF-κB signaling is also interdicted by SseK1 and SseK3. These two effectors modify the death domains of members of this pathway with their arginine glycosyltransferase activity [92,93]. The effector AvrA, on the other hand, inhibits NF-κB and JNK signaling with acetyltransferase activity toward MAPKKs [87,88].

The virulence plasmid is harbored by many strains of non-typhoidal serovars. It contains the *spv* operon, which is composed of four genes that are important for systemic spread. SpvB inhibits NF-κB activity by downregulating IKKβ in addition to depolymerizing actin and disrupting tight junctions [108]. SpvC is a phosphothreonine lyase. It irreversibly dephosphorylates Erk1/2, p38 and JNK [109]. Since phosphothreonine lyase activity has only been observed in pathogens, SpvC and its homologs could serve as attractive narrow-spectrum antibiotic targets. SpvC is dispensable for survival within macrophages and the gastrointestinal tract but is required for non-typhoidal *Salmonella* to cause bloodstream infections of mice and humans, as is SpvD [22,127,128]. SpvD inhibits the nuclear transport of NF-κB p65 with its cysteine hydrolase activity. Interestingly, the degree to which it affects NF-κB signaling is affected by a serovar-specific polymorphism [67,111]. 

Inflammatory responses are thought to control infections by activating microbial killing mechanisms and regulating host cell death. Relatively little attention has been paid to how they prevent the migration of infected cells. The host presumably benefits from localizing its phagocytes to an area of infection to better fight it and does not want to inadvertently spread it. *Salmonella* neutralizes this infection control mechanism with anti-inflammatory effectors to facilitate the colonization of systemic sites. Most notably, SpvC is required for reverse transmigration and likely also for migration through the lymphatic system [22]. The same is probably true of many other, if not all, virulence factors with anti-inflammatory activity, which needs to be tested.

SopD is a bifunctional virulence factor that at times can provoke inflammation and, enigmatically, at others, suppresses it. It has GTPase activity towards Rab8 that induces inflammation but can also dampen it by displacing it from its guanosine dissociation inhibitor [98]. SopD and SopB activate a PI3K–PKB–mTOR pathway that is dependent on RAB8, producing IL-10, which counters inflammation [98,126,129,130]. The effector SteE/SarA/GogC reprograms a cytokine receptor’s effector to induce anti- rather than pro-inflammatory genes [131,132]. SteE targets STAT3, which the host normally uses to attenuate inflammation [133,134]. Interestingly, it involves the host kinase GSK3 that acts on SteE and not the Janus kinases [131,132].

## 3. Outbreaks in the Tropics

### 3.1. Background

The collective action of the pro- and anti-inflammatory effectors of *Salmonella* are largely responsible for the estimated 3.4 million cases of invasive disease with non-typhoidal serovars of *Salmonella* per year globally [135]. The most impacted area of the world is the tropics. The serovars most frequently responsible include *S*. Dublin and especially *S*. Enteritidis and *S*. Typhimurium [135]. Between 2008 and 2018, *Salmonella* was responsible for more bloodstream infections that resulted in hospitalization in Africa and Asia than any other pathogen by a considerable margin [136]. 

### 3.2. Invasive S. Typhimurium in Sub-Saharan Africa

In 2009, multi-locus sequence typing of conserved housekeeping genes identified a novel *S*. Typhimurium sequence type (ST), ST313 [137]. It is of particular concern as it is responsible for epidemic invasive disease in Sub-Saharan Africa. One of the most apparent distinguishing features of invasive lineages of non-typhoidal *Salmonella* is the loss of genes involved in exacerbating gut inflammation and thriving in this environment [138]. While genome degradation associated with host specialization and systemic disease has been well studied, it is interesting to consider that the pseudogenization of genes involved in gut colonization may not be critical for allowing sepsis. These genes may merely be dispensable. Perhaps allelic differences in the genes that regulate inflammation are more determinative of whether a strain can disseminate extraintestinally. Numerous alleles of various type III effectors are associated with either gastrointestinal or systemic disease [68]. Thus, of particular interest are the 700 single nucleotide polymorphisms that separate ST313 from ST19 [139]. ST19 is primarily associated with gastroenteritis globally but has extensive synteny with ST313 [139]. These 700 single nucleotide polymorphisms may be critical to understanding the ability of ST313 to cause bloodstream infections.

ST313 is composed of three distinct, yet tightly clustered, lineages termed L1, L2 and L3 [140]. L1 and L2 are distinct, but tightly clustered, differing from each other by 455 single nucleotide polymorphisms [137]. The success of African ST313 lineages is attributed in part to the multi-drug resistance phenotype that many of the strains express. L2 is resistant to chloramphenicol, which was once the drug of choice for treating sepsis in Malawi and played a role in the displacement of L1 by L2 [140]. Chloramphenicol was replaced with ciprofloxacin and then third-generation cephalosporins for the treatment of sepsis in sub-Saharan Africa, to which some strains of L2 have recently acquired resistance [140]. L3 appeared in 2016 in Malawi. Despite its recent emergence, it is a phylogenetic intermediate between L1 and L2 with a unique genome degradation pattern [140]. The ST313 L2 representative strain D23580 has 23 pseudogenes versus the 6 found in the ST19 prototypical strain SL1344 [140].

ST313 is not geographically restricted to sub-Saharan Africa, as originally thought. Isolates from this ST are also present in the UK and South America, where they are primarily associated with gastroenteritis [141]. Comparative analyses of the UK, South American and Sub-Saharan African ST313 isolates are especially informative in understanding the invasiveness of ST313 in Africa. ST313-UK genomes show the same degradation as D23580 with only three characterized genes, SrfH/SseI, *lpxO* and *macB*, functional in ST313-UK but not in D23580. The dominant allele of *srfH* that is functional in ST313-UK may suppress the deadhesion of infected cells in a way that inhibits systemic dissemination [24]. Interestingly, the dominant allele is only present in strains that are primarily associated with gastroenteritis [68]. LpxO modifies lipid A in a way that may alter infection dynamics whereas MacB is a macrolide efflux pump [142,143]. Also important in understanding the potential for sepsis with ST313 in Africa are the BTP1–BTP5 prophages and plasmid borne multi-drug resistance loci found in sub-Saharan ST313 but lacking in ST313-UK. 

In addition to pseudogenization and differences in the alleles of functional genes, extragenic regions must also be considered. African ST313 strains contain a single nucleotide polymorphism in the *pgtE* promoter that increases its expression versus an ST19 background that enhances resistance to human complement and increases virulence in chickens [144]. A transcriptomic study revealed that 677 genes and small RNAs are differentially expressed between the ST313 L2 representative strain D23580 and ST19 strain 4/74 [145].

### 3.3. Invasive Salmonella Enteritidis in Sub-Saharan Africa

*Salmonella enteritidis* is a major cause of enterocolitis in industrialized countries, whereas in Africa, it frequently causes invasive disease. The global epidemic clade is a generalist and is weakly invasive. It is associated with the intensive poultry industry in developed countries. There are two related clades that are a frequent cause of sepsis in Africa, which originated in the sub-Saharan region of this continent [146]. Fifty-seven predicted genes associated with the prophage ΦSE20 are unique to the global epidemic clade, as are an additional 39 genes. The Central/Eastern African clade contains 77 predicted genes that are not found in other clades, with 33 of these on the virulence plasmid and another 40 harbored in a Fels-2 like prophage region [146]. There are only 15 distinct predicted genes in the West African clade, of which 11 were associated with the virulence plasmid. The virulence plasmid of the African clades is significantly larger than that of the global epidemic clade at 90 Kb versus 58 Kb, including the genes for multiple drug resistance [146]. As with *S.* Typhimurium ST313, there is evidence of significant genome degradation in the African clades, including 42 hypothetically disrupted genes involved in gut colonization [147]. Of additional interest are the 363 genes with non-synonymous single nucleotide polymorphisms as such amino acid-altering mutations that can change the course of infection [21,67,68]. 

### 3.4. S. Typhi H58

In sub-Saharan Africa and Southeast Asia, *S*. Typhi haplotype 58 (H58) has been a major source of outbreaks and epidemics of systemic *Salmonella* disease [148,149,150,151]. These organisms first emerged in 1987 in India and then spread throughout South Asia, and then globally, in the ensuing years. The genetic determinants of the success of this haplotype remain to be fully identified and characterized but are undoubtedly due in part to its multi-drug resistance phenotype. H58 strains can resist chloramphenicol, ampicillin, tri-methoprim-sulfamethoxazole and fluoroquinolones, as well as, alarmingly, third-generation cephalosporins. The extensive multi-drug resistance and spread of H58 strains globally poses a major public health problem.

### 3.5. Host Susceptibility to Extraintestinal Dissemination

To comprehensively understand *Salmonella* bloodstream infections, in addition to the microbial factors, one must consider what characteristics of the host render one susceptible to bacteremia. The first lineage of ST313 in sub-Saharan Africa arose in tandem with the HIV pandemic. In fact, over 95% of African adults with invasive non-typhoidal *Salmonella* disease are co-infected with HIV [152,153]. In African children, predisposing factors include not just HIV co-infection but also malaria and malnutrition [154,155]. 

Numerous genes associated with immune responses have been implicated in the systemic dissemination of non-typhoidal and typhoidal *Salmonella.* These studies were carried out in the murine model of *Salmonella* infection or were observed in humans with immunodeficiencies. Interestingly, the two sets of host susceptibility factors, one for enteric fever and the other for invasive non-typhoidal *Salmonella* disease, do not completely overlap. This suggests that perhaps typhoidal and non-typhoidal *Salmonella* have different mechanisms and routes of extraintestinal dissemination.

All four of the Toll-like receptors that recognize *Salmonella* pathogen-associated molecular patterns assist the host in controlling *Salmonella* infection in mice. Similarly, cases of rare primary immunodeficiencies in humans have implicated TLR signaling in deterring invasive *Salmonella* disease [156,157]. Moreover, a TIRAP missense mutation that attenuates TLR signal transduction is associated with susceptibility to bacteremia with non-typhoidal *Salmonella*. Cases of enteric fever were extremely uncommon, suggesting there are differences in how typhoidal and non-typhoidal *Salmonella* cause bacteremia [158]. TLR polymorphisms were associated with enteric fever in one population study but not in others [159,160,161]. 

Key to preventing invasive *Salmonella* disease is the production of pro-inflammatory cytokines in response to TLR signaling, including IL-12 and IFN-γ, which activate an infected phagocyte. This is illustrated by the set of rare primary immunodeficiencies referred to as MSMD, caused by mutations in eight autosomal genes and two X-linked genes: IFNGR1, IFNGR2, STAT1, IL12B, IL12RB1, IRF8, ISG15, TYK2, and CYBB and IKBKG. MSMD renders one susceptible to disseminated disease with both non-typhoidal *Salmonella* and poorly pathogenic mycobacteria, as do IFN-γ autoantibodies. A human genome-wide association study identified a locus in the STAT4 region that influences IFN-γ production in natural killer cells in a manner that renders carriers susceptible to bacteremia with non-typhoidal *Salmonella* [162]. Interestingly the susceptibility allele protects from a variety of autoimmune disorders, revealing a trade-off between the ability to effectively combat infectious disease and not enable inflammatory ones [162]. There is some evidence for a link between enteric fever and IL-12 and IFN-γ, but it is not as clear as it is with invasive non-typhoidal *Salmonella* [163,164,165]. In addition to IL-12, TLR activation will also cause phagocytes to produce TNF, which acts synergistically with IFN-γ. TNF receptor-deficient mice and ones that are depleted for TNF are more susceptible to the dissemination of non-typhoidal *Salmonella* [166,167]. The murine deficiencies IL-1ß, IL-18, NLRP3-NLRC4 or caspase 1 also result in increased susceptibility to early systemic dissemination of *Salmonella* [168,169,170]. 

In a human genome-wide association study, patients with MHC class II deficiency were susceptible to non-typhoidal *Salmonella* infection while resistance to enteric fever was conferred by a HLA-DRB1 single nucleotide polymorphism [171]. This highlights that not only the innate immunity but also the adaptive immunity mediated by T cells is critical for deterring invasive *Salmonella* disease.

### 3.6. Persistent Infections

Extraintestinal *Salmonella* dissemination sometimes results in a chronic infection. These infections are asymptomatic but are a major public health concern for two reasons. First, they can contribute to the development of gallbladder cancer [172]. Second, the bacteria can be intermittently shed for, in some cases, the life of the carrier, and thus serve as a reservoir from which the pathogen can spread to new hosts and from which new genotypes can emerge [173]. *S*. Typhi biofilm formation on the surface of gallstones is associated with chronic infections [172]. In persistent infections, the bacteria primarily reside within macrophages. *Salmonella* changes the immune status of the infected macrophages with SteE, which induces a non-inflammatory polarization that counteracts TNF-mediated pathogen restriction [174,175]. *Salmonella* metabolically reprograms these cells to promote bacterial persistence, by, among other things, inducing them to express a high level of the host transcription factor PPARδ that increases the availability of glucose [176]. Systemic sites are thus a highly desirable niche for the bacteria with reduced competition and are rich in nutrients that they are well equipped to exploit. While less than 5% of infections result in chronic carriage, this may be the driving force behind the ability of *Salmonella* to cause systemic disease. 

Genes within *Salmonella* pathogenicity island-1, -2, -3, -4, -5 and -6, as well as the integrated phages GIFSY-1 and GIFSY-2, among others, are important for persistence in a mouse model of long-term *S.* Typhimurium systemic infections [177]. Of particular interest is the *Salmonella* pathogenicity island-2-associated effector *srfH*/*sseI* [177]. *srfH* has been reported to bind the host proteins filamin, TRIP6 and IQGAP-1 [20,21,23,178]. It was more definitively demonstrated to deamidate the heterotrimeric G protein Gαi2, resulting in its persistent non-polarized activation [179]. One of the effects of this is the reduction of directed dendritic cell migration, that other reports suggest could result from increased adhesion [23,179]. This loss of the directed migration of infected dendritic cells enhances the long-term colonization of mice in a chronic carrier model of disease [23]. This may be attributable to a loss of directed phagocyte movement along T cell chemoattractive gradients in systemic tissue [23]. SrfH mutants also hyper-disseminate through lymphatic vessels to the mesenteric lymph nodes, perhaps by facilitating the deadhesion/migration of infected phagocytes [20,23,24]. The possibility that mutants may also allow antigen sampling dendritic cells to dissociate from the basal face of the gastrointestinal epithelium more efficiently in the reverse transmigration pathway needs to be tested [20,21,23,24]. 

### 3.7. The Role of SrfH/SseI in Extraintestinal Dissemination

*srfH* was the first gene for which different naturally occurring alleles were shown to affect virulence [21]. The alleles identified influence whether the infection is confined to the gastrointestinal tract or spreads to systemic tissue early in infection [21,68]. Intriguingly, some alleles of *srfH*, as with *sopD2*, possesses seemingly opposed activities. The C-terminus of *srfH* alleles harbored by serovars primarily associated with gastrointestinal disease in humans suppresses deadhesion. The alleles predominant in serovars and specific isolates which can cause disseminated disease have single nucleotide polymorphisms in or near the catalytic sites in the C-terminus and/or polymorphisms in the N-terminus, which seem to promote deadhesion, perhaps by suppressing an inflammatory response [20,21,68,180]. However, some alleles possess the C-terminal domain that promotes adhesion and an N-terminal domain that appears to inhibit it [20,21,23,24,68,179]. More work is required to uncover how the spatiotemporal activities of such alleles of *srfH* and/or its host targets are regulated during infection. The *srfH* allele of hyper-invasive sequence type 313 isolates is a pseudogene that intriguingly contains the carboxyl terminal catalytic residues that allow for Gαi2 deamidation. It does not, however, contain any of the compensatory polymorphisms in other regions of the genes that are present in other invasive strains that at times appear to counter the effect of the carboxyl region on adhesion [23,24,68]. The different phenotypes reported for *srfH* in various reports are likely due to the use of of different alleles of this intriguing effector [20,21,23].

## 4. Conclusions

Bloodstream infections with *Salmonella* are a growing public health threat that are particularly relevant in the tropics. The significance is compounded by the emergence of multi-drug resistance and the lack of a licensed vaccine for invasive non-typhoidal *Salmonella*. How the pathogen transitions from colonizing the gut to spreading to the blood is incompletely understood. It seems to involve a switch from inducing and exacerbating host inflammation to attenuating it. More work is needed to better understand how the pathogen shifts from one phase of disease to the other. This could provide us with new therapeutic intervention opportunities for compartmentalizing infections, thereby greatly reducing their chance of fatality. 

Recent evidence points to the role of dampening the host’s inflammatory response in allowing for the deadhesion and migration of infected phagocytes to allow systemic dissemination [22,67]. This is an area in need of additional research. The cytokine macrophage migration inhibitory factor has pluripotent effects but was initially described as a soluble factor that potently inhibits the migration of macrophages [181]. Perhaps there is an important link between the function originally ascribed to this inflammatory cytokine and the systemic spread of intracellular pathogens. The host likely possesses redundant mechanisms for localizing phagocytes to an area of infection, all of which must be neutralized for a pathogen to exploit these cells as vehicles for dissemination to deeper tissue. There are, in fact, scores of enteropathogenic type III effectors that possess anti-inflammatory activities, some of which are required for systemic dissemination [66,93]. Perhaps a major function of them, considering that most are not required for intracellular survival or to interdict host cell death pathways, is the regulation of infected host cell adhesion and migration [66,77,93]. This role of the inflammatory response has been largely overlooked even though it may by critical to reducing lethal infections. An additional area in need of investigation is the possibility of potentially complex epistatic interactions within and between the suites of pro- and anti-inflammatory effectors. 

It is interesting to consider that enteropathogens may exploit undescribed routes of extraintestinal dissemination. *Salmonella* pathogenicity island-1 is required for efficient invasion of the epithelium and CD18 is required for reverse transmigration. The observation that neither are necessary for *Salmonella* to travel from the gut to systemic circulation suggests that there may be pathways that remain to be discovered [70]. The invasins PagN and Rck could be involved in an undescribed route as they promote the invasion of epithelial cells independently of *Salmonella* pathogenicity island-1 [182,183]. 

One of the drawbacks of some studies that seek to assess the relative contributions of one particular pathway to systemic disease is that when a specific route is blocked and no defect is observed, more of the inoculum may simply go through other routes than it normally does. This possibility was elegantly addressed with *Yersinia*, by infecting mice in which all pathways were available with a pool of strains that harbored molecular tags, and tracking them though the course of infection. That study concluded that this enteropathogen disseminated directly to the spleen and liver from a replicating pool of bacteria in the lumen of the gut, independently of the lymphatic system [78]. Perhaps a similar approach could be tried with *S.* Typhimurium.

The possibility of hybrid modes of dissemination also needs to be explored. For example, lumenal bacteria could invade enterocytes, traffic to the basolateral side, exit, be taken up by phagocytes in the lamina propria and then induce them to reverse transmigrate into the bloodstream. This would provide an explanation for the observation that lamina propria CX3CR1^+^ phagocytes in BALB/c mice do not seem to extend dendrites into the lumen of the gut, but bacteria including *Salmonella* can still rapidly arrive in the bloodstream inside of such cells [20,184]. Bacteria could also potentially trigger the reverse transmigration of infected phagocytes through the high endothelial venules associated with Peyer’s patches following M cell invasion and destruction. This possibility would, interestingly, involve causing the infected cells to transmigrate in the conventional apical to basal direction through the lymphatic endothelium, and then reverse transmigrate in the basal to apical direction through the blood vascular endothelium. Clues as to how non-typhoidal *Salmonella* colonizes the bloodstream are provided by the requirements for *Salmonella* pathogenicity island-2 and in particular, the type III effectors SpvC and SpvD. These effectors will undoubtedly be the subject of intensive future research [22,127,185,186]. Both effectors are anti-inflammatory and are necessary for *Salmonella* to cross the mucosal barrier of mice [22,127,185,186]. They are also important for bacteremia with non-typhoidal serovars in humans [22,110,127,128,187].

The most immediate public health challenges associated with invasive *Salmonella* disease are providing treatment for those infected with malaria and/or HIV, as well as nutrition to the malnourished. Those afflicted that go untreated are immunocompromised in a fashion that renders them highly susceptible to invasive non-typhoidal *Salmonella*. Also urgent is the need for the development of new anti-microbials which are difficult for *Salmonella* to overcome. New antibiotic targets are needed as most molecules conventionally considered ‘drugable’ are targeted by existing drugs [188,189]. Unfortunately, multi-drug resistant strains of *S*. Typhi have been commonplace for some time [190,191]. *S*. Typhi strains resistant to chloramphenicol, ampicillin and trimethoprim have been responsible for numerous outbreaks [192]. These strains have become so commonplace that chloramphenicol was withdrawn as the first-line drug for typhoid fever and replaced with fluoroquinolones and third generation cephalosporins [193]. However, in India, typhoidal strains that are resistant to both nalidixic acid and ciprofloxacin have become endemic, producing instances of nearly intractable typhoid fever [194]. Such strains have also been reported in the US and UK, reflecting the emergence of a global problem [194]. The situation with multi-drug resistance is equally dire, with invasive non-typhoidal *Salmonella* with African isolates displaying extensive multi-drug resistance. Given the rapid rate at which *Salmonella* evolves resistance to traditional anti-microbials, there is a pressing need to develop new drugs, preferably ones which the microbe will be unable to quickly evolve ways to overcome. Also urgent in the case of invasive non-typhoidal *Salmonella* is the need for a safe and effective licensed vaccine.

## Figures and Tables

**Table 1:** Major pro-and anti-inflammatory effectors.

Effector	Full Name	Typhimurium	Typhi	Secreted by	Biochemical Activity	Host Binding Partners	Functions	References
AvrA	Anti-virulence gene A	+	−	SPI-1 and SPI-2	Acetyltransferase	ERK2, MKK4, MKK7, p53	Inhibits NF-κB signaling, inflammation and apoptosis	[87,88]
GogA	Gisy-one gene A	+	−	SPI-1 and SPI-2	Zinc metalloprotease	NF-κB p65	Inhibits NF-κB signaling	[77]
GogB	Gifsy-one gene B	+	−	SPI-1 and SPI-2	Adaptor protein	SKP1, FBXO22	Inhibits NF-κB signaling	[89]
GtgA	Gifsy-two gene A	+	−	SPI-1 and SPI-2	Zinc metalloprotease	Class II NF-κBs (p65, RelB and cRel)	Inhibits NF-κB signaling	[90]
PipA	Pathogencity island protein A	+	+	SPI-2	Zinc metalloprotease	NF-κB p65	Inhibits NF-κB signaling	[90]
SlrP	*Salmonella* leucine rich repeat protein	+	+	SPI-1 and SPI-2	E3 ubiquitin ligase	Thioredoxin, SNRPD2, ERdj3, UbcH5b	Inhibits the release of IL-1ß and attenuates inflammasome activation	[91]
SseK1	*Salmonella* secreted effector K 1	+	−	SPI-1 and SPI-2	Glycosyltransferase	FADD, TRADD, Rab1, Rab5, Rab11	Inhibits TNF-alpha-stimulated NF-κB signaling and necroptosis	[92,93]
SseK2	*Salmonella* secreted effector K 2	+	−	SPI-2	Putative glycosyltransferase		Inhibits TNF-alpha-stimulated NF- kappaB signaling and necroptosis	[94]
SseK3	*Salmonella* secreted effector K 3	+/−	+	SPI-2	Glycosyltransferase	TNFR1, TRAILR, TRIM32	Inhibits TNF-alpha-stimulated NF-κB signaling and necroptosis	[92]
SipA	Salmonella invasion protein A	+	+	SPI-1		Caspase-3, F-actin, T-plastin, syntaxin8	Disrupts tight junctions, among others	[75,76]
SopA	*Salmonella* outer protein A	+	-	SPI-1	E3 ubiquitin ligase	TRIM56, TRIM65, UbcH5a, UbcH5c, UbcH7, HsRMA1, Caspase-3	Invasion, PMN migration	[95]
SopB	*Salmonella* outer protein B	+	+	SPI-1	Phosphoinositide phosphatase	Cdc42	Activates Rho-family GTPase GEFs	[96,97]
SopD	*Salmonella* outer protein D	+	+	SPI-1 and SPI-2	GAP and GEF	Rab8 and Rab10	Invasion, inflammation and fluid secretion	[98]
SopE	*Salmonella* outer protein E	+/−	+	SPI-1	GEF	Cdc42, Rac1 and Rab5	Inflammation via NF-κB signaling	[99,100,101]
SopE2	*Salmonella* outer protein E 2	+	+	SPI-1	GEF	Cdc42 and Rac1	Inflammation via NF-κB signaling	[99,100,101]
SopF	*Salmonella* outer protein F	+	−	SPI-1	ADP ribosyltransferase	ATP6V0C, ARF1 PDK1	Attenuates intestinal epithelial cell inflammation, allowing systemic dissemination among other things	[102]
SptP	*Salmonella* protein tyrosine phosphatase	+	+	SPI-1	GAP and tyrosine phosphatase	Cdc42, Rac1, VCP, vimentin, cSrc, NSF and Syk	Inactivates Cdc42 and Rac1, inhibits ERK	[103,104,105]
SpvB	*Salmonella* plasmid virulence B	+	−	SPI-1 and SPI-2	ADP-ribosyltransferase	G-actin	Depolymerizes actin, inhibits NF-κB signaling, disrupts intestinal epithelial barrier, promotes systemic dissemination and disease	[57,106,107,108]
SpvC	*Salmonella* plasmid virulence C	+	−	SPI-1 and SPI-2	Phosphothreonine lyase	ERK1/2, p38 and JNK	Suppresses pro-inflammatory signaling by inhibiting MAPKs, promotes reverse transmigration	[22,109,110]
SpvD	*Salmonella* plasmid virulence D	+	−	SPI-1 and SPI-2	Cysteine hydrolase PKN1, Ube2D	Exportin-2?	Inhibits NF-κB signaling	[111]
SspH1	*Salmonella* secreted protein H1	+/−	−	SPI-1 and SPI-2	E3 ubiquitin ligase	PKN1, Ube2D	Ubiquitinates host kinase PKN1 for degradation, suppresses NF-kappaB activation, inhibits androgen steroid receptor and macrophage activation	[112,113]
SspH2	*Salmonella* secreted protein H2	+	+	SPI-2	E3 ubiquitin ligase	Nod1, SGT1, UbcH5- Ubiquitin	Activates Nod1 signaling	[114,115]
SteA	*Salmonella* translocated effector A	+	+	SPI-1 and SPI-2	Adaptor protein	GSK3α/β, STAT3	Transcriptional reprogramming toward anti-inflammatory phenotype	[116]

## Data Availability

No new data were created or analyzed in this study. Data sharing is not applicable to this article.
